# Dopamine Adaptations as a Common Pathway for Neurocognitive Impairment in Diabetes and Obesity: A Neuropsychological Perspective

**DOI:** 10.3389/fnins.2017.00134

**Published:** 2017-03-28

**Authors:** Dana M. Small

**Affiliations:** ^1^The John B Pierce LaboratoryNew Haven, CT, USA; ^2^Department of Psychiatry, Yale University School of MedicineNew Haven, CT, USA

**Keywords:** dementia, diabetes, obesity, dopamine, cognition, executive function, memory, associative learning

## Abstract

Evidence accumulates linking obesity and diabetes with cognitive dysfunction. At present the mechanism(s) underlying these associations and the relative contribution of diet, adiposity, and metabolic dysfunction are unknown. In this perspective key gaps in knowledge are outlined and an initial sketch of a neuropsychological profile is developed that points toward a critical role for dopamine (DA) adaptations in neurocognitive impairment secondary to diabetes and obesity. The precise mechanisms by which diet, metabolic dysfunction, and adiposity influence the DA system to impact cognition remains unclear and is an important direction for future research.

## Neurocognitive impairments in type 2 diabetes (T2D)

T2D is associated with cognitive decline, brain dysfunction, and dementia (Biessels et al., 2014; Koekkoek et al., 2015; Stoeckel et al., 2016). One recent study estimated that the combined overall relative risk for dementia is 73% higher in people with, compared to without T2D, indicating that between 1 in 10 and 1 in 15 incidences of dementia may be attributable to T2D (Biessels et al., 2014). Although glucose intolerance is diagnostic of T2D, a recent systematic review of 86 papers examining T2D and cognition only reported a weak association between glycaemia, and cognition (Geijselaers et al., 2015) and there is even less evidence for an association with other measures of peripheral glucose regulation and cognitive function (e.g., insulin concentration, insulin action, insulin resistance) (Geijselaers et al., 2015). Thus, although T2D is by definition associated with altered glucose metabolism, it is not clear that altered glucose metabolism contributes to cognitive change. The mechanism behind the link between cognitive dysfunction and T2D is therefore not clear.

## Neurocognitive deficits may arise from chronic conditions associated with T2D

The majority of human studies linking T2D to cognitive decline are performed in older individuals with long-standing diagnoses of diabetes (Stoeckel et al., 2016). This poses a problem for interpreting the pathophysiology of the link between T2D and cognition because individuals with chronic T2D exhibit a number of pathologies associated with cognitive decline such as damage to the blood brain barrier (BBB), neuroinflammation (Banks et al., 2012; Steculorum et al., 2014), cerebral atrophy, and small vessel disease (Biessels and Reijmer, 2014; Akrivos et al., 2015; Ramos-Rodriguez et al., 2016; Stranahan et al., 2016). The co-occurrence of these pathologies that are secondary to diabetes has led to controversy over whether it is T2D (Biessels and Reagan, 2015) or complications arising from T2D that leads to cognitive decline (De Felice and Ferreira, 2014). To rule-out confounds associated with the secondary complications of T2D it will be informative to study cognition in populations free from other chronic conditions and in populations prior to the onset of T2D. For example, it would be informative to characterize neurocognition in youth before and after the onset of prediabetes, since this population will be free from other chronic conditions that could influence cognitive function.

## It is unknown if neurocognitive deficits are associated with T2D or adiposity or both

Perhaps the most important limitation of the current literature is the failure to disentangle effects of metabolic dysfunction on cognition from those of adiposity and diet. Obesity has been associated with altered brain structure and function in animal models and in metabolically and neurologically healthy adults and children (Elias et al., 2003; Reinert et al., 2013; Hsu and Kanoski, 2014; Yau et al., 2014; Bocarsly et al., 2015), while diets high in saturated fat and cholesterol are correlated with compromised cognitive flexibility and processing speed in pre-pubertal children after adjusting for age, sex, socioeconomic status, IQ, VO_2max_, and BMI (Khan et al., 2015b). Consumption of a high fat diet (HFD) can also negatively impact brain and brain function well-before obesity onset. For example, in animal models hypothalamic insulin resistance is observed following acute exposure to HFD before changes in adiposity occur (Clegg et al., 2011) and impaired performance on hippocampal-dependent tasks is observed after only 72-h access to a HFD when animals have actually lost weight, presumably due to neophobia (Kanoski and Davidson, 2010). These findings suggest that obesity can impact cognition independently from metabolic disease and that diet can impact metabolic function and cognition independently of obesity.

To date, studies have rarely taken obesity and diet into account when examining the relationship between T2D and cognition. For example, patients with T2D exhibit reduced activity in the default mode network (Musen et al., 2012), which has been associated with a wide range of neurological conditions and cognitive impairments (Browndyke et al., 2017; Contreras et al., 2017; Jockwitz et al., 2017; von Rhein et al., 2017) but BMI, which was higher in T2D, was not accounted for. Similarly, the putative confound of glucose intolerance is often not considered when examining the relationship between obesity and cognition. For example, a prospective study examining the impact of obesity on cognition excluded participants for many medical conditions likely to influence cognition, including stroke, dementia, myocardial infarction, and atrial fibrillation but NOT diabetes (Gunstad et al., 2010). They did however, include “glucose intolerance” in their mixed model regression analyses and found that this variable was related to cognitive impairments that also correlated with their adiposity measures (waist-hip ratio). In another study deficits in executive function and declarative memory were observed in 38 middle-aged adults with insulin resistance but without T2D compared to 54 age, gender, education but NOT BMI matched controls. Since the insulin resistance group had significantly higher BMI these deficits may be equally attributable to BMI (Bruehl et al., 2010).

Failure to account for confounds between diet, obesity, and metabolic dysfunction also pervade the animal literature. Rats prone to develop diabetes upon HFD are often used as a model of T2D (Levin and Routh, 1996). These models have been associated with deficits on the water maze (Li et al., 2002; Stranahan et al., 2008b), object recognition test (Stranahan et al., 2008a), contextual cue conditioning (Grillo et al., 2011), and discrimination and reversal learning (Kanoski et al., 2007, 2010). HFD has also been shown to increase inflammatory cytokines and impair neuroplasticity and learning and memory in the hippocampus (Erion et al., 2014). The extent to which adiposity or insulin resistance contributed to these observations is not known. However, impaired cognition is also observed with the streptozotocin (STX)-induced diabetic model, which impairs insulin production without increasing adiposity or requiring a high fat diet, indicating that metabolic dysfunction alone is sufficient to impair cognitive function (Stranahan et al., 2008a).

Importantly, when more than one variable is measured interactions between adiposity, diet, and impaired glucose tolerance are revealed. In obese humans without T2D, insulin sensitivity mediates the relationship between working memory-related activation in the right parietal cortex and BMI (Gonzales et al., 2010), while brain insulin action is selectively impaired in the prefrontal cortex in overweight and obese, but not diabetic adults compared to their lean counterparts (Kullmann et al., 2015), highlighting interactions between adiposity and glucose tolerance on brain function.

In summary, the relative contribution of diet, impaired glucose tolerance, and adiposity to neurocognitive impairment is largely unexplored and unknown.

## Characterization of glycemia

Another factor clouding the association between T2D and cognitive impairment is the use of a variety of methods to characterize glycemia, each of which reflect different, and sometimes independent, aspects of glucose metabolism (Geijselaers et al., 2015). Insulin sensitivity can be measured using a variety of techniques. HbA_1c_, which reflects the mean glucose concentration over a period of 8–12 weeks is the most frequently used measure. Fasting blood glucose concentrations are also frequently measured, which reflect nocturnal hepatic gluconeogenesis that is influenced by hepatic insulin sensitivity, but a recent review found that studies often fail to report whether measurements are taken at the same time of day (Geijselaers et al., 2015). Other measures include post-prandial glucose concentrations, reflecting insulin secretory responses and HOMA-IR to measure insulin resistance. HbA_1c_ shows the strongest association to insulin resistance, followed by post-prandial measures. Fasting glucose, by contrast, seems to be unrelated to cognitive performance (Geijselaers et al., 2015). Interestingly, one study found that insulin resistance was related to declarative memory whereas HbA_1c_ was associated with executive dysfunction (Bruehl et al., 2010), hinting at the possibility that the different measures are associated with distinct pathophysiological effects on the brain and highlighting the need for more comprehensive measures of glucose metabolism.

## Neurocognitive impairments may be related to central rather than (or in addition to) peripheral impairments in glucose tolerance

Insulin receptors are widely distributed in the brain, with the highest concentrations in the olfactory bulb, hypothalamus, cerebral cortex, cerebellum, and hippocampus (Havrankova et al., 1978; van Houten et al., 1979). Brain-specific deletion of the insulin receptor in mice results in glycogen synthase kinase 2 beta activation resulting in hyperphosphorylation of tau protein, a hallmark of early Alzheimer's Disease (AD) (Schubert et al., 2004). There is also evidence from animal studies that disrupted central insulin and insulin-like growth factor-1 (IGF-1) signaling may lead to disrupted neurotransmitter (e.g., dopamine) and astroglial cell function, brain endothelial cell function involved in formation and regulation of BBB, mitochondrial metabolism and oxidative stress, clearance of Aβ and/or amyloid fibrils, cholesterol synthesis in the brain (important for myelination and membrane function), glucose and lipid metabolism in select regions of the brain, and regulation of central energy balance, which could relate to both metabolic and neurocognitive dysfunction (Brüning et al., 2000; Convit et al., 2003; Schubert et al., 2004; Suzuki et al., 2010; Kleinridders et al., 2014; Stouffer et al., 2015). While these data suggest a likely role for central insulin resistance in impaired neurocognitive function, it is important to note that central insulin resistance has a complicated relationship with peripheral glycemic control (Ketterer et al., 2011). Central insulin resistance is thought to result from a combination of impaired insulin receptor signaling and decreases in the transport of insulin across the BBB (Banks et al., 2012), which can occur secondary to peripheral glucose intolerance (Niswender et al., 2003). Conversely, central insulin signaling contributes to peripheral glucose regulation (Brüning et al., 2000; Heni et al., 2014) to create a dynamic brain-gut axis regulating glucose metabolism. Importantly, however, central insulin resistance can occur independently from peripheral impairments in glucose tolerance. Post-mortem studies of brain tissue from patients with AD but not T2D, reveal disrupted brain insulin signaling (De Felice and Ferreira, 2014; Yarchoan and Arnold, 2014). Accordingly, treatment with intranasal insulin, which results in direct insulin transport from the nasal cavity to the CNS via intraneuronal and extraneuronal pathways (Reger and Craft, 2006), improves cognition in patients with (Reger et al., 2008; Craft et al., 2012) and without (Hallschmid et al., 2007, 2008) dementia. These findings underscore the importance of concurrent measures of peripheral and central insulin resistance.

One promising avenue for future research is in using intranasal insulin in combination with neuroimaging methodologies and neuropsychological testing to assess the role of central insulin resistance in neurocognition (Tschritter et al., 2006; Ketterer et al., 2011; Grichisch et al., 2012; Kullmann et al., 2013, 2015; Heni et al., 2014, 2016). For example, intranasal insulin decreases the blood oxygen dependent (BOLD) response in the hypothalamus and PFC increases BOLD response in the striatum (Schilling et al., 2014) and insular cortex (Heni et al., 2012) and increases brain energy levels (Jauch-Chara et al., 2012). Critically, these effects are blunted in obesity (Tschritter et al., 2006) with evidence that hypothalamic insulin resistance is driven by visceral fat and frontal insulin resistance by peripheral insulin sensitivity (Kullmann et al., 2015). Collectively, these findings indicate a complex relationship between peripheral glucose control and central insulin resistance and they raise the possibility that central insulin resistance contributes to cognitive impairment in concert with, or independently from peripheral impaired glucose tolerance.

## Neurocognitive deficits may be domain-specific and differentially influenced by diet, adiposity, and metabolic dysfunction

Although obesity and T2D are occasionally associated with global measures of brain atrophy (Enzinger et al., 2005; Gunstad et al., 2010; Raji et al., 2010; Brooks et al., 2013) and cognitive decline (Liang et al., 2014), many studies suggest that executive function is the domain most affected in both adults (Gunstad et al., 2007; Sabia et al., 2009; Fitzpatrick et al., 2013) (Volkow et al., 2009) and children (Convit et al., 2003; Reinert et al., 2013; Liang et al., 2014). For example, negative correlations are observed between BMI and performance on tasks of executive function but not episodic verbal memory (Volkow et al., 2009) with BMI negatively, and executive performance positively, correlated with baseline prefrontal glucose metabolism. Similarly, a recent meta-analysis of 21 studies concluded that obesity is associated with impairments in decision-making, planning and problem solving with less evidence for associations with verbal fluency and learning and memory (Fitzpatrick et al., 2013). Correspondingly, structural changes (Enzinger et al., 2005; Pannacciulli et al., 2006; Raji et al., 2010; Fotuhi et al., 2012; Bocarsly et al., 2015) and reduced brain connectivity (Musen et al., 2012) are observed in the parietal and prefrontal cortex (PFC), which are critical for executive function.

There is also strong evidence from animal work that HFD produces hippocampal insulin resistance (Biessels and Reagan, 2015) and damage (Hsu and Kanoski, 2014), resulting in impaired hippocampal-dependent cognitive functions (Kanoski and Davidson, 2011). Likewise, hippocampal atrophy is observed in obese humans (Raji et al., 2010) and altered hippocampal white matter connectivity is found in T2D (van Bussel et al., 2016). However, there are inconsistent findings with respect to alterations in hippocampal-dependent episodic memory tasks (Fitzpatrick et al., 2013). For example, significant deficits in working memory and in reinforcement learning are observed in the absence of episodic learning and memory impairment, in obese vs. healthy weight adults that are matched for age, gender, education, and IQ (Coppin et al., 2014). In contrast, impaired episodic memory and decreased hippocampal volume is observed as a function of glucose tolerance (Convit et al., 2003) and intranasal insulin increases the functional connectivity between the hippocampus and PFC in people with T2D (Zhang et al., 2015). Other studies report hippocampal-dependent impairments as a function of saturated fat intake (Francis and Stevenson, 2011) and central, but not whole body adiposity (Khan et al., 2015a). Collectively these data suggest that episodic memory may be affected by diet and metabolic dysfunction while being unrelated to BMI and whole body obesity.

**Figure 1:**
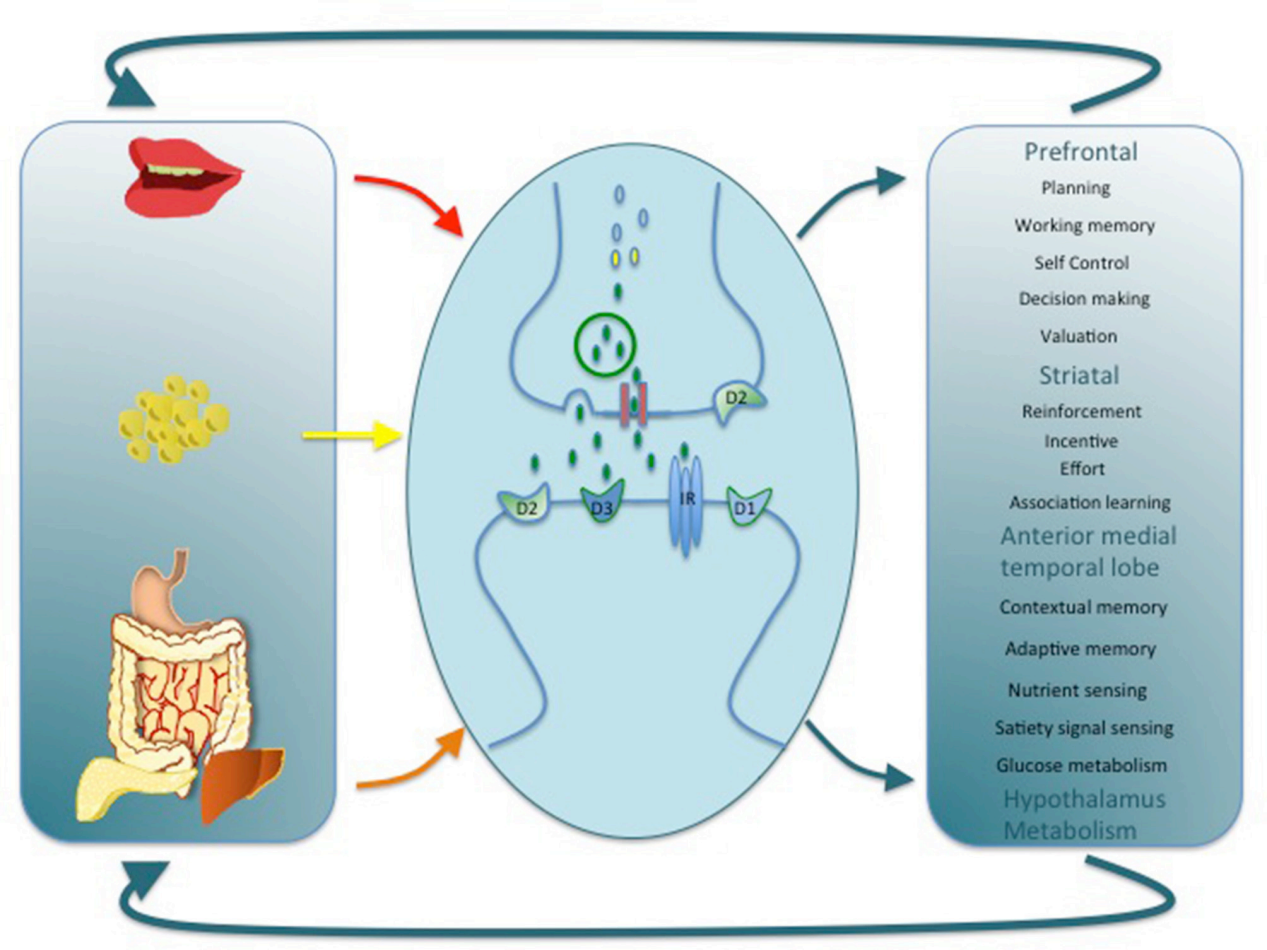
**This cartoon depicts alterations in DA signaling as a common link by which diet, adiposity, and metabolic dysfunction might impact cognition, motivation, and energy balance**. A variety of mechanisms at the cellular and molecular level could support this association by regulating pre and post-synaptic DA receptor expression, DA synthesis, release, and reuptake. Alterations at any level may in turn have a wide impact on brain function and provide a parsimonious explanation for a number of dysfunctions associated with obesity and T2D.

This emerging neuropsychological profile provides an important insight into the pathophysiological mechanism that gives rise to neurocognitive impairment in obesity and T2D. Brain functions associated with diabetes and obesity tend to rely upon DA signaling (Figure 1). For example, the dopaminergic fronto-striatal loop plays a well-known role in working memory, cognitive flexibility, reinforcement learning, and incentive motivation (Frank and Fossella, 2011). It is also critical for response inhibition, the failure of which is associated with addictive like behaviors including overeating (Lokken et al., 2009; Maayan et al., 2011; Lee et al., 2013; Guo et al., 2014; Zhao et al., 2016). Finally, a role for DA in memory via a projection from the ventral tegmental area to the hippocampus has been described (Shohamy and Adcock, 2010). As reviewed above, these DA-dependent cognitive processes are altered in obesity/T2D, raising the possibility that a common pathway by which diet, adiposity and metabolic dysfunction might coalesce to impact cognition is by producing alterations in the DA system (Figure 1). Interestingly, although DA adaptations are considered integral in the development of compulsive behaviors and alterations in reward sensitivity they are not typically considered as a potential mechanism behind other neurocognitive complications in T2D and obesity.

There are consistent findings in the animal literature that HFD, and adiposity alter DA signaling at the cellular, and molecular levels (Anderzhanova et al., 2007; Johnson and Kenny, 2010; van de Giessen et al., 2012, 2013; Sharma and Fulton, 2013; Tellez et al., 2013; Cansell et al., 2014; Adams et al., 2015; Woods et al., 2016), as well as mounting evidence for altered DA signaling in human obesity, especially reflected in changes in DA receptor density, (Wang et al., 2001, 2011; Dunn et al., 2010; Steele et al., 2010; Eisenstein et al., 2013, 2015; Guo et al., 2014; Cosgrove et al., 2015; Horstmann et al., 2015; Karlsson et al., 2015; Caravaggio et al., 2015b; Dang et al., 2016; Gaiser et al., 2016). Additionally, there is evidence that central and peripheral insulin resistance might impact DA function. Central insulin signaling, acting through the downstream modulator Akt, is a potent modulator of DA transporter (DAT) activity, which fine-tunes DA signaling at the synapse (Kleinridders et al., 2015), demonstrating a pathway by which central IR could influence the DA system. Insulin administration also suppresses DA release by clearing DA from the synapse and concomitantly reducing the rewarding properties of food (Figlewicz and Sipols, 2010). Likewise, peripheral insulin sensitivity is associated with reduced endogenous DA levels (Murzi et al., 1996; Caravaggio et al., 2015a) and peripheral glycemia with PFC-striatal-hippocampal functional connectivity (Page et al., 2013). Thus, diet, adiposity, and insulin resistance could each impact DA signaling with the potential for additive effects and interactions. Future work aiming to disambiguate the unique and interacting effects will therefore be an important step toward understanding neurocognitive impairment in T2D and obesity.

## Summary

In summary, a consensus is emerging that obesity and diabetes are accompanied by cognitive impairments and brain dysfunction and that at least some of these effects are secondary to their onset. Multiple mechanisms have been proposed to underlie these associations but at present it is unclear which mechanism, or mechanisms, are critical. Also unclear is whether diet, obesity and metabolic dysfunction have distinct and/or converging pathways to neurocognitive impairment. However, work emerges to suggest that all three factors may influence DA signaling, which is provocative since the cognitive impairments that characterize diabetes and obesity uniformly rely upon the integrity of the DA system. It is therefore proposed that adaptations in DA signaling secondary to diet, adiposity and metabolic dysfunction underlie much of the neurocognitive impairment observed in diabetes and obesity.

## Author contributions

The author confirms being the sole contributor of this work and approved it for publication.

## Funding

This work was supported by NIH NCI R01CA180030 awarded to DMS and Ivan de Araujo.

### Conflict of interest statement

The author declares that the research was conducted in the absence of any commercial or financial relationships that could be construed as a potential conflict of interest.
